# From Wage Dissatisfaction to Union Expectations: The Mediating Role of Union Perception Among Nurses

**DOI:** 10.1111/jan.70580

**Published:** 2026-03-19

**Authors:** Salih Tosun, Gökçe Cerev, Doğa Başar Sariipek

**Affiliations:** ^1^ Department of Political Science and Public Administration, Faculty of Economics and Administrative Sciences Balıkesir University Balıkesir Türkiye; ^2^ Department of Labor Economics and Industrial Relations, Faculty of Political Sciences Kocaeli University Kocaeli Türkiye

**Keywords:** mixed method, nursing, union expectation, union membership, union perception, wage satisfaction

## Abstract

**Aim:**

This study examines the relationships between wage satisfaction, union perceptions, expectations, and union membership among Turkish nurses, identifying factors associated with union participation.

**Design:**

Sequential explanatory mixed‐methods design.

**Methods:**

This two‐phase study (January–July 2023) included: (1) a quantitative survey of 210 nurses assessing wage satisfaction, union perceptions, and expectations, analysed using regression and PROCESS Macro (Model 4); and (2) qualitative interviews with 22 nurses, including 15 with union leadership experience, analysed through thematic analysis using MAXQDA.

**Results:**

Quantitative findings indicated that wage satisfaction was associated with nurses' perceptions of unions but did not independently predict union expectations. The study hypothesises that union perception functions as a key mediating mechanism, translating wage dissatisfaction into expectations for union action. Qualitative findings supported this pattern, showing that although wage dissatisfaction was widespread, nurses' expectations were primarily shaped by perceptions of unions' transparency, political independence, democratic participation, and representational capacity rather than by wages alone.

**Conclusions:**

Union participation among nurses is influenced by both economic conditions and normative evaluations of unions. While wage dissatisfaction provides an important contextual background, expectations and engagement are mainly driven by perceptions of union credibility, fairness, and representational effectiveness.

**Implications for Profession and/or Patient Care:**

Nursing unions should prioritise transparent governance, democratic participation, and political independence to enhance trust, member engagement, workforce stability, and quality of care.

**Impact:**

This study addresses persistent wage dissatisfaction alongside declining union membership and trust. The findings demonstrate that union engagement depends not only on economic dissatisfaction but also on perceived representational fairness. The results are particularly relevant for nursing unions, professional organisations, and policymakers aiming to strengthen union legitimacy and workforce engagement in healthcare systems.

**Reporting Method:**

Compliant with COREQ guidelines and mixed‐methods reporting standards.

**Patient or Public Contribution:**

No patient or public contribution.

## What Does This Paper Contribute to the Wider Global Clinical Community?

- Highlights the central role of normative trust and representation in shaping union engagement among nurses.
- Demonstrates how economic dissatisfaction alone is insufficient to generate union expectations.
- Offers insights relevant to healthcare systems facing workforce dissatisfaction and union legitimacy challenges.

## 1 Introduction

Nursing is widely recognised as a cornerstone of healthcare systems, characterised by high emotional labour, professional responsibility, and substantial physical demands (Zhao et al. 2022). The job satisfaction of nurses and the quality of care they provide are affected by multiple interrelated factors, including wage satisfaction, working conditions, organisational structures, and psychosocial elements such as organisational justice, professional value, and commitment (Aloisio et al. 2021; Penconek et al. 2021; Lee et al. 2023).

Among these factors, wage satisfaction stands out as a central dimension, as it encompasses not only financial sufficiency but also perceptions of recognition, respect, and fairness (Lu et al. 2005; Torlak and Göktepe 2024). Cross‐national evidence points to marked differences in remuneration levels. In 2021, hospital nurses in Türkiye earned a median annual income of USD 29,177 (PPP), a figure that surpasses median earnings in some OECD member states yet remains below the OECD average (OECD 2023). Further studies show that during periods of structural transformation in healthcare systems, wage satisfaction plays a critical role in shaping employee motivation and organisational performance (Göktepe et al. 2020; Abella 2022). Taken together, these conditions form an important context for examining how wage satisfaction informs nurses' perceptions of unions and their expectations of union roles, both in instrumental and normative terms.

Even though wages have risen in real terms since 2010, recurring economic instability and sustained inflation—most notably after the pandemic—have substantially reduced the practical value of these increases. As a result, concerns related to fair remuneration, staff retention, and professional out‐migration have become more pronounced (Tosun and Cerev 2023; Aslan 2023; Gülşen et al. 2025; WHO 2020). Within institutionalised labour systems, trade unions continue to operate as central channels through which employees express claims over pay, job security, and collective representation (Fiorito and Padavic 2022; Dierkes et al. 2024). Nevertheless, previous research consistently shows that high levels of union membership do not necessarily correspond to individual satisfaction or trust in unions (Düzenli and Demir 2022; Şafak et al. 2023). Notably, studies also indicate that perceptions of inadequate wages do not always diminish expectations directed at unions, a pattern often linked to the ongoing symbolic and relational functions that unions fulfil beyond immediate economic outcomes (Wiegand and Bruno 2018).

## 2 Research Aim and Questions

The present study employs a sequential mixed‐methods design to explore the relationship between wage satisfaction and union membership behaviours among nurses in a single public hospital system and its affiliated campuses. Quantitative analysis models associations between wage satisfaction, union perceptions, and expectations, followed by qualitative inquiry into nurses' interpretations of these relationships.

Central Research Question:

- How is nurses' wage satisfaction related to their perceptions and expectations of unions, and how are these factors associated with union membership?

Sub‐research Questions:

- RQ1 (Quantitative Phase): To what extent is wage satisfaction associated with nurses' perceptions of unions and their expectations from union membership?
- RQ2 (Qualitative Phase): How do nurses interpret the relationship between wage dissatisfaction and union perceptions and expectations, and how does this relate to their union membership decisions?

## 3 Background

Wage satisfaction is commonly treated as a matter of income level, yet empirical and conceptual work suggests that it is better understood as a broader evaluative judgement that also involves perceptions of fairness, recognition, and professional worth (Lu et al. 2005; Torlak and Göktepe 2024). Within healthcare settings, dissatisfaction with pay has been linked to a range of adverse work‐related outcomes, including lower job satisfaction, diminished organisational commitment, and stronger intentions to leave the profession. These associations appear particularly salient among nurses, whose roles are characterised by sustained emotional engagement alongside intensive physical demands (Aloisio et al. 2021; Penconek et al. 2021).

At the micro level, evaluations of pay tend to emerge through comparative and expectation‐based processes. Nurses assess their wages not only in absolute terms, but also in relation to perceived fairness, occupational status, and economic security. Early theoretical contributions help to clarify these dynamics. Equity Theory emphasises perceived imbalances between individual inputs and received rewards (Adams 1963), while Discrepancy Theory focuses on the distance between expected and realised earnings (Lawler 1971). Subsequent conceptual refinements have expanded this view by recognising that wage satisfaction encompasses multiple dimensions, including base pay, pay progression, hierarchical positioning, and fringe benefits (Heneman and Schwab 1979). Research in labour‐intensive healthcare contexts has since demonstrated that these pay evaluations are closely intertwined with perceptions of distributive and procedural justice, as well as with broader assessments of professional prestige (Williams et al. 2006; Khatak 2015).

At the mezzo level, unions operate as more than bargaining instruments focused narrowly on wage outcomes. They also function as institutional reference points through which employees form judgements about fairness, voice, and organisational legitimacy (Hyman 2001; Kelly 2012). Employees' evaluations of unions tend to centre on whether unions can effectively safeguard job security, defend social and employment rights, and offer representation that is both fair and accountable. Democratic decision‐making processes and transparency in union practices play a particularly important role in shaping these assessments (Bryson et al. 2004; Turner and D'Art 2012; Fidan and Yetiş 2018). When unions are perceived as open and independent, they are more likely to sustain members' engagement; conversely, doubts regarding transparency or political alignment often erode trust and participation.

Expectations directed at unions reflect prospective judgements about what unions are likely to deliver in the future. These judgements typically extend beyond wage‐related outcomes to include fringe benefits, employment security, and the quality of representation in interactions with employers and policymakers (Hammer and Avgar 2005; Kochan et al. 2019). Research on expectations suggests that unmet anticipations can weaken satisfaction and commitment over time, even in contexts where formal representation remains strong (Roese and Sherman 2007). Taken together, these dynamics indicate that sustained union engagement depends not only on tangible outcomes but also on members' broader evaluations of organisational competence, ethical governance, and representational credibility.

At the macro‐institutional level, broader structural and political factors shape union–member relationships. Membership involves an implicit exchange in which employees expect material or representational returns, while unions are expected to act in a fair and reliable manner (Ward and Berno 2011; Kaynak 2021; Doğan and Sökmen 2021). Although classical labour economics has framed unions mainly as instruments for wage improvement (Freeman and Medoff 1984), empirical research shows that links between wage satisfaction and union‐related attitudes are neither linear nor one‐directional (Tremblay and Roussel 2001; Bryson et al. 2004; Currall et al. 2005; Nelson et al. 2008; Hammer and Avgar 2005). In Türkiye, where unionisation in public health and social services exceeds 75% but individual satisfaction remains relatively low (Şafak et al. 2023; ÇSGB 2024), union participation reflects a combination of economic considerations and legitimacy‐related factors, including trust, representational quality, and political neutrality (Hyman 2001; Kelly 2012; Fiorito and Padavic 2022; Dierkes et al. 2024). This perspective underlines the value of examining wage satisfaction, union perceptions, and expectations together within the institutional context of Turkish healthcare.

## 4 Method

### 4.1 Research Design

**FIGURE 1:**
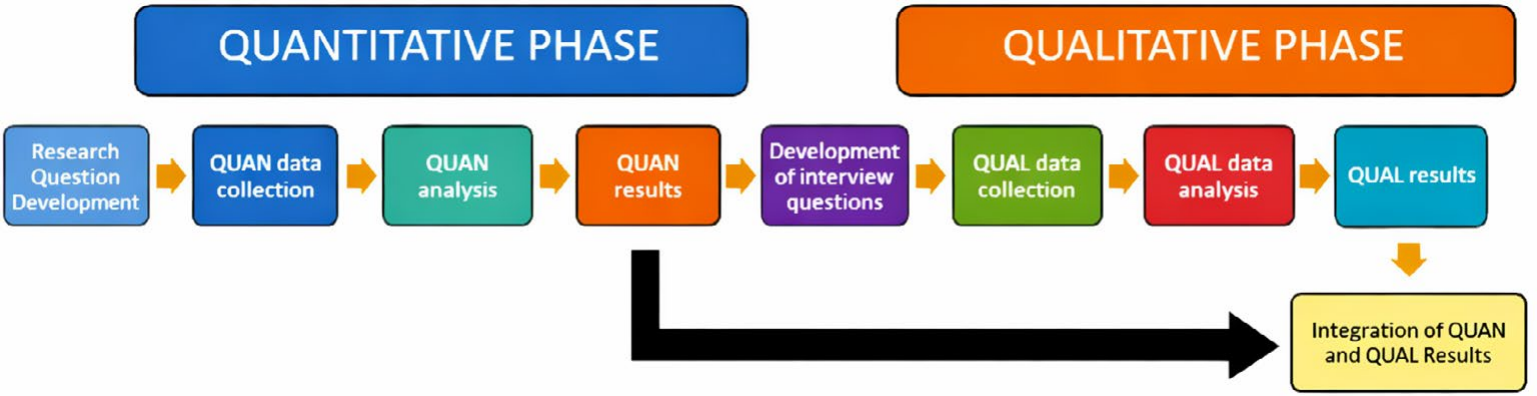
Sequential explanatory mixed‐method research design.

This study adopted a sequential explanatory mixed‐methods design (Creswell 2009), beginning with a cross‐sectional face‐to‐face survey, followed by individual semi‐structured interviews. This design enabled the exploration of unexpected patterns or gaps identified in the quantitative data through deeper qualitative inquiry (Doyle et al. 2009). Both research phases were interconnected from the outset, with the qualitative interview questions developed in response to findings from the quantitative phase to ensure coherence. Data from both phases were integrated after separate analyses were completed. In this design, the quantitative phase served as the core component, while the qualitative phase provided contextual insight and explanation (Figure 1). This method was chosen due to the availability of validated measurement tools and well‐established variables in the literature, offering a systematic and rigorous framework for addressing the research questions. Source: Hayes et al. (2015).

### 4.2 Participants and Sampling

This study employed a cross‐sectional sample of nurses working at Balıkesir City Hospital. As of May 2023, the hospital employed approximately 680 nurses. City hospitals in Türkiye are public, tertiary‐level healthcare institutions located in each province, providing comprehensive specialised care to the local population. The sample included nurses from various wards and clinics, not limited to a specific department.

Following institutional approval, the recruitment process involved direct, face‐to‐face outreach within the clinical units. This on‐site approach allowed for a clear explanation of the study's objectives, the guarantee of anonymity, and the strictly voluntary nature of the project during standard working hours. Because the study offered no external incentives, participation relied solely on genuine interest. Consequently, the data collection only proceeded once each nurse provided formal, informed consent.

All participants held a 4‐year undergraduate nursing degree and had acquired the necessary professional competencies. Eligibility for the quantitative phase hinged on three specific requirements: active employment in clinical services during the data collection period, a minimum workload of five shifts over a 2‐week span, and a clear commitment to voluntary participation. The selection process intentionally excluded those on temporary leave or assigned to strictly administrative duties.

Out of 220 nurses who voluntarily completed the questionnaire, data from 210 were deemed valid and included in the analysis. The remaining 10 questionnaires were excluded because they contained incomplete responses. This was primarily because the surveys were administered within the nurses' work environments. In high‐demand units such as emergency departments or outpatient clinics, additional workload and urgent patient care needs occasionally resulted in unanswered items or the inability to complete the questionnaire. Removing these incomplete surveys ensured that the analysis was based on reliable and consistent data.

A post hoc power analysis using GPower indicated a statistical power of 0.89 for the ANOVA, with an effect size of 0.47 and a 5% margin of error. According to Cohen (1988), research power (1−*β* error) reflects the likelihood of detecting statistically significant results, and a power level of 0.80 or higher is generally considered strong (Kline 2023). These results support the adequacy of the sample size for the analyses conducted.

The qualitative phase utilised purposive sampling (Patton 2002), drawing participants from the initial survey pool based on specific professional profiles. Initial selection focused on individuals with direct experience in union management or governance who were willing to sit for in‐depth interviews. However, as fieldwork progressed, it became clear that male nurses dominated the existing union leadership structures. To counter this imbalance and broaden the study's perspective, the sampling strategy expanded to intentionally recruit female nurses, regardless of their union membership status. This adjustment ensured the final sample provided the information‐rich cases necessary to address the research questions from a more equitable standpoint.

Although qualitative research does not require strict rules regarding sample size, Namey et al. (2016) suggest that 8 to 16 interviews are generally sufficient. In this study, 22 in‐depth interviews were conducted, and data saturation was achieved. No participants withdrew or declined participation after the data collection process began.

### 4.3 Data Collection Tools and Procedures

The quantitative phase of the study utilised a face‐to‐face questionnaire, which included demographic information along with measures of wage satisfaction, union perception, and expectations from unions. Data collection took place between March and June 2023.

The Wage Satisfaction Scale, which consists of 24 items across three sub‐dimensions, assesses the level of satisfaction nurses feel with their total monthly income in relation to their professional activities. The subcomponents of this scale include organisational commitment, competence, and organisational policies. Scores for each subscale range from 1 to 5, with higher scores indicating greater satisfaction with wages and lower scores reflecting lower satisfaction (Yıldırım and Demirel 2015).

The Union Perception Scale comprises 24 items, including 5 reverse‐coded items, divided into three sub‐dimensions that measure nurses' attitudes toward unions, as well as their views on the functionality and representativeness of unions. The subcomponents include union, membership, and the state‐employer relationship. Each subscale is scored on a 1 to 5 scale, with higher scores indicating stronger positive perceptions of unions and lower scores indicating weaker perceptions. A total mean score above or near 2.77 indicates a moderately positive perception (Tekin and Tüfekçi 2015).

The Union Expectation Scale consists of 24 items in three sub‐dimensions, evaluating nurses' expectations from unions, their views on the benefits unions provide to their members, and the services they expect from union membership. The subcomponents of this scale are economic, social, and political expectations. Scores range from 1 to 5, with higher scores reflecting higher expectations from unions and lower scores indicating lower expectations (Tekeş 2010).

All scales demonstrated acceptable to good model fit according to standard fit indices (Schermelleh‐Engel et al. 2003). The internal reliability of the measures was evaluated using Cronbach's alpha coefficient, with an overall alpha value of 0.98. The subscales for pay satisfaction had alphas ranging from 0.92 to 0.96, union perception subscales ranged from 0.82 to 0.94 (overall alpha of 0.87), and union expectation subscales ranged from 0.70 to 0.85 (overall alpha of 0.93). Importantly, all scales had been previously validated and tested for reliability in the same language as the participants, and content validity was supported through expert review by an academic with expertise in qualitative research in the social sciences, as well as a pilot test to ensure clarity and cultural appropriateness.

The qualitative phase involved semi‐structured interviews conducted between May and June 2023. From the quantitative phase, 40 participants expressed interest in participating. Participants were invited in groups of 10 via WhatsApp messages, which included information and consent forms to be completed upon confirmation. As responses were received, new groups of 10 participants were selected and invited using the same process. This approach followed a maximum variation sampling strategy, ensuring representation of key characteristics identified in the quantitative phase, such as union membership, representation, and management roles.

The nursing profession in Türkiye constitutes a critical context for examining participation patterns in union activities. The profession is historically female‐dominated due to its development around care‐oriented labour, which has been culturally and socially associated with women. Modern nursing institutionalised in the early 20th century through Volunteer Patient Care courses, and educational programmes were initially open only to women. The 1954 Nursing Act legally prohibited men from entering the profession, a restriction that was lifted only in 2007 (Tosun et al. 2025). Consequently, nursing has long remained numerically dominated by women. However, leadership and decision‐making positions in health sector unions are predominantly occupied by men, reflecting a paradox in which high female representation in the profession contrasts with limited female participation in union governance (Keleş 2018).

To account for this structural imbalance and capture a diverse range of experiences, the qualitative sample included not only union representatives and unionised nurses but also female nurses, both with and without union membership. The interview questions were crafted based on the analysis of the quantitative data, aiming to explore deeper insights into the research variables and address unexpected findings (Table 1). Interviews lasted between 40 and 60 min, were digitally recorded, and transcribed verbatim for analysis.

**TABLE 1:** Interview questions.

**Interview schedule**
*Preliminary information/introduction*
Can you share how long you have been working as a nurse and how your views on trade unions have evolved throughout your career?
Are you currently a member of a trade union? If yes, what motivated you to join? If not, what are your reasons for not joining?
*Wage Satisfaction*
How do you feel about the adequacy of your salary within your organisation? Do you think your salary reflects the work you do?
What financial or non‐financial aspects of your job bring you the most satisfaction?
How does your salary influence your work motivation and overall commitment to your profession?
Did your level of wage satisfaction play a role in your decision to join the union?
*Union Perception*
What is your opinion on the role of trade unions in the workplace? How effective do you think unions are in terms of job security, rights protection, and wage policies?
Do you believe your union is successful in negotiating wages on your behalf?
How do you evaluate the impact of unions on wage increases or social benefits?
How do you perceive your colleagues' views on the union?
*Union Expectation*
What are your primary expectations from trade unions (e.g., wage increases, job security, social rights)?
Do you feel your union is meeting these expectations? Why or why not?
What do you hope to gain most from your union membership?
Do you think unions are doing enough to enhance wage satisfaction and improve working conditions?
How can trade unions better contribute to your job satisfaction?
*General*
In your opinion, how should unions work to improve wage satisfaction and working conditions?
What changes would you recommend to make union activities more effective?

After the initial thematic analysis, the study incorporated a partial member‐checking stage. Preliminary interpretations were shared with a purposively selected subgroup of participants (*n* = 8 of 22), comprising unionised nurses, non‐unionised nurses, and union leaders. The process remained partial by design, as it did not include the full qualitative sample. Nurses' shift‐based schedules and the intensity of clinical workload constrained opportunities for follow‐up engagement, limiting validation to rest periods or leave days and reducing overall availability. Participants reviewed the interpretations for accuracy, clarified meanings, and commented on the developing themes. The analysis integrated this feedback to strengthen credibility and interpretive coherence.

### 4.4 Ethical Considerations

Ethical approval for the study was granted by Kocaeli University Social and Human Sciences Ethics Committee (Approval No: 2021/14–12) on November 16, 2021. The study was further approved by the Balıkesir Provincial Directorate of Health, Ministry of Health of Türkiye on February 18, 2022, under decision number E‐51829602‐604.01.02. Comprehensive information about the study was provided to potential participants at the start of each phase. Verbal informed consent was obtained from all participants, and their confidentiality rights were fully assured.

### 4.5 Data Analysis

The quantitative and qualitative data sets were analysed independently and then integrated to offer a comprehensive assessment. The quantitative analysis focused on exploring the relationships between wage satisfaction, union perceptions, and union expectations, while the qualitative analysis provided an in‐depth exploration of nurses' personal experiences with these issues.

#### 4.5.1 Quantitative Data Analysis

Statistical analyses were performed using IBM SPSS Statistics version 26 and the PROCESS Macro v4.2 (Hayes 2017). All nurses participating in the study completed face‐to‐face questionnaires. Prior to data analysis, the distributional normality of the data, the presence of extreme values, and the reliability indices were assessed. Skewness and Kurtosis values were used to examine the normal distribution of variables.

In the data analysis, descriptive statistics were presented in terms of frequencies, means, and percentages. We examined the mediating effect with Hayes' (2017) PROCESS Macro, selecting Model 4. To interpret the relationships between the study variables, we applied Pearson correlation and linear regression analyses. We set the level of statistical significance at *p* < 0.05.

#### 4.5.2 Qualitative Data Analysis

Inductive thematic analysis was employed for data analysis and interpretation (Sundler et al. 2019). The audio recordings were transcribed verbatim, and the data were carefully reviewed to generate initial codes based solely on the information provided by the participants, which were then grouped into themes. The analysis followed the six steps outlined by Braun and Clarke: (1) familiarising with the data, (2) generating codes, (3) searching for themes, (4) reviewing themes, (5) defining and naming themes, and (6) producing the report (Braun and Clarke 2006). Steps three through five were carried out independently by the researchers, followed by a collaborative review to refine the themes and confirm that data saturation had been achieved. Data saturation was assessed using the method proposed by Guest et al. (2020). According to this approach, a Base Size of 4, Run Length of 2, and a New Information Threshold of 5% were applied, and it was observed that no new themes emerged after the 15th participant (Guest et al. 2020). The analyses, organised around the research questions, were conducted by the third author and the first author using MAXQDA Analytics Pro 2024 software. In the final stage, all authors discussed the results and reached a consensus. This approach enriched the conceptual depth of the themes and ensured a thorough and rigorous analysis.

### 4.6 Integration Strategy

In mixed‐methods research, integration is defined as the deliberate process of combining different methodological sequences to generate more comprehensive insights than those produced by a single approach, while preserving the unique contributions of each approach (Moran‐Ellis et al. 2006; Greene 2007; Creswell and Plano Clark 2007). Recent methodological discussions emphasise that integration is not merely a procedural step but a theoretically grounded practice that enhances the interpretive power of findings (Fetters et al. 2013). In this study, integration occurred after identifying the qualitative themes, which were subsequently used as an explanatory framework to interpret and contextualise the emerging quantitative findings. This sequential explanatory integration aligns with Creswell and Plano Clark's (2007) typology, where qualitative results are mobilised to provide depth and meaning to quantitative outcomes, ensuring complementarity and strengthening the overall validity of interpretations.

### 4.7 Rigour and Trustworthiness

In an explanatory mixed methods design, it is crucial to minimise threats to validity. To ensure rigour in this study, a sufficient sample size and reliable measurement tools were utilised in the quantitative phase, while participants for the qualitative phase were selected based on key findings from the quantitative data. In the qualitative data collection process, priority was given to participants from the quantitative phase as well as individuals with experience in union management and representation. The data were analysed independently and later subjected to comparative evaluation (Creswell and Plano Clark 2007).

Four key criteria were applied in the qualitative phase to enhance reliability: validity, transferability, dependability, and confirmability (Lincoln and Guba 1985). Data collection was concluded once data saturation was achieved. To ensure transferability, the research environment and participants were described in detail, and the COREQ checklist was used to further strengthen reliability (Tong et al. 2007). To strengthen validity and reliability, we implemented a systematic member‐checking strategy. Following the initial thematic analysis, we presented preliminary findings to eight purposively selected participants from the main qualitative sample of 22, including unionised and non‐unionised nurses as well as union leaders. These participants were asked to review the accuracy of interpretations, provide clarifications, and offer additional insights. Feedback from this process was then integrated into the final analysis, which led to the refinement of several themes and sub‐themes, ensuring that the thematic structure accurately reflected participants' experiences. Additionally, one of the researchers has a professional background in nursing, which facilitated contextual understanding and interpretation of participants' experiences. This dual approach ensured that the results genuinely reflected participants' perspectives and minimised potential researcher bias. Finally, direct quotations from participant statements were included in the analysis to reinforce confirmability.

## 5 Results

In accordance with the sequential explanatory design, the findings from the dominant quantitative phase will be presented first, followed by the qualitative results, which provide a deeper understanding and interpretation of these findings (Creswell 2009).

### 5.1 Participant Characteristics

The demographic characteristics of the 210 nurses who participated in and completed the quantitative study are presented in Table 2. Of the participants, 72.4% were female, and 67.6% were married. Regarding age distribution, 47.8% were 30 years old or younger, 22.0% were between 31 and 40 years old, and 32.4% were over 41 years old. In terms of professional position, 97.6% worked in clinical units within health services, while 2.4% worked in administrative services. Regarding work experience, 47.6% had 11 years or more of experience. As for their family income‐expenditure balance, 41.0% reported their income was less than their expenses, 49.5% stated it was equal, and 9.5% indicated their income exceeded their expenses. Finally, 83.3% of the participants were union members, while 16.7% were not (Table 2).

**TABLE 2:** Characteristics of the participants (Quantitative Phase).

Descriptive characteristics	Variables	*n* (210)	%
Age	30 and below	96	47.8
	31–40	46	22.0
	41 and above	68	32.4
Gender	Male	58	27.6
	Female	152	72.4
Marital status	Married	142	67.6
	Single	68	32.4
Service class	Clinical health services	205	97.6
	Administrative services	5	2.4
Professional experience (Years)	10 years or less	110	52.4
	11 years and above	100	47.6
Family income and expenditure balance	Income less than expenditure	86	41.0
	Income equal to expenditure	104	49.5
	Income more than expenditure	20	9.5
Union membership	Union member	175	83.3
	Non‐union member	35	16.7

In the qualitative phase, 22 nurses (10 men and 12 women) who held leadership and representative roles in various unions within the health service sector were interviewed. The participants' ages ranged from 29 to 55, and their professional experience spanned from 10 to 35 years. Their tenure in the union ranged from 1 to 16 years. The participants represented 7 different unions, with 10 serving as workplace representatives (some unions had more than one representative), 3 as union branch presidents, and 2 as city representatives, of whom 3 were union members and the remaining 4 were non‐union nurses (Table 3).

**TABLE 3:** Characteristics of the participants (Qualitative Phase).

Participants	Age	Gender	Time spent in the profession	The union (s)he works for	Position in the trade union	Term of office in the trade union
P‐1	32	Female	10	Health and Social Workers Union (Sağlık‐Sen)	Workplace Representative	4
P‐2	32	Female	19	Health and Social Services Workers Union of Türkiye (Sağlık Çalışanları Sendikası)	Workplace Representative	1
P‐3	30	Male	12	Health and Social Workers Union (Sağlık‐Sen)	Workplace Representative	4
P‐4	35	Male	15	Turkish Health and Social Services Public Workers Union (Türk Sağlık‐Sen)	Workplace Representative	7
P‐5	36	Male	12	Young Health and Social Service Workers' Union (Genç Sağlık‐Sen)	Workplace Representative	5
P‐6	38	Male	15	Biz Health and Social Service Workers' Union (Biz Sağlık‐Sen)	Workplace Representative	1
P‐7	38	Male	18	Turkish Health and Social Services Public Workers Union (Türk Sağlık‐Sen)	Workplace Representative	3
P‐8	47	Male	27	Health and Social Workers Union (Sağlık‐Sen)	Workplace Representative	8
P‐9	46	Female	25	Nurses and All Health Professionals Union (HEP‐SEN)	Workplace Representative	16
P‐10	29	Male	10	Turkish Health and Social Services Public Workers Union (Türk Sağlık‐Sen)	Workplace Representative	8
P‐11	46	Male	25	Health and Social Workers Union (Sağlık‐Sen)	Union Branch President	12
P‐12	45	Female	26	Young Health and Social Service Workers' Union (Genç Sağlık‐Sen)	Union Branch President	4
P‐13	54	Male	33	All Health and Social Service Workers' Union (Tüm‐Sen)	City Union Representative	2
P‐14	29	Female	10	Innovative Health and Social Service Workers' Union (Yeni Sendika)	City Union Representative	1
P‐15	55	Male	35	Services Public Workers Union (Türk Sağlık‐Sen)	Union Branch President	10
P‐16	35	Female	11	Turkish Health and Social Services Public Employees Union (Türk Sağlık‐Sen)	Union member	9
P‐17	29	Female	6	Health and Social Workers Union (Sağlık‐Sen)	Union member	5
P‐18	42	Female	19	Young Health and Social Service Workers' Union (Genç Sağlık‐Sen)	Union member	15
P‐19	32	Female	8	Non‐union member	Non‐union member	—
P‐20	37	Female	11	Non‐union member	Non‐union member	—
P‐21	32	Female	7	Non‐union member	Non‐union member	—
P‐22	30	Female	5	Non‐union member	Non‐union member	—

### 5.2 Quantitative Results

Table 4 presents the reliability results of the scales and their subdimensions used in the study, along with the number of items, means, and standard deviations for each scale. Skewness and Kurtosis analyses indicated that all values ranged between +1.5 and −1.5, demonstrating that the data were normally distributed (Tabachnick and Fidell 2013). The internal consistency coefficients of the scales ranged from 0.87 to 0.98. Since reliability coefficients above 0.70 are considered acceptable in social sciences research, it can be concluded that all scales employed in the study possess sufficient reliability (Büyüköztürk et al. 2013). Analysis of the mean scores of the scales applied to nurses revealed that wage satisfaction was low, with a mean score of 1.93 on a 5‐point Likert scale. Conversely, union perception was at a moderate level (mean score = 2.97), while union expectation was relatively higher (mean score = 3.28). These findings suggest that nurses are dissatisfied with their wages, hold ambivalent perceptions of unions, and maintain moderate expectations regarding the services that unions should provide (Table 4).

**TABLE 4:** Descriptive statistics of the scales (normality distribution, reliability coefficients, means, and standard deviations).

Scales	Subdimensions	Number of items	Mean/SD		*α*	Skewness and Kurtosis	
**Wage Satisfaction Scale**	Total Score	**24**	**1.9387**	**0.88765**	**0.980**	**1.054**	**0.878**
Subdimensions	Organisational Commitment	4	1.9060	0.93626	0.925	1.084	0.881
	Competence	5	1.9648	0.95792	0.929	0.973	0.364
	Institutional Policies	15	1.9387	0.88457	0.966	1.044	0.872
**Union Perception Scale**	Total Score	**21**	**2.9710**	**0.62259**	**0.872**	**0.146**	**0.172**
Subdimensions	Union	13	2.7667	0.93745	0.945	−0.199	−0.359
	Membership	5	3.7362	0.90121	0.871	−0.188	−0.695
	Government and Employer	3	2.5810	0.96403	0.823	−0.010	−0.517
**Union Expectation Scale**	Total Score	**24**	**3.2752**	**0.80742**	**0.939**	−0.**603**	**0.721**
Subdimensions	Economic	6	3.4238	0.97882	0.856	−0.541	−0.073
	Social	16	3.2687	0.85372	0.917	−0.492	0.356
	Political	2	3.0254	0.81610	0.728	−0.243	0.192

*Note:* Bold values indicate Total Score.

Table 5 presents the results of three regression models predicting nurses' union perception and union expectation. Statistical analysis within the first model identifies a clear positive path between wage satisfaction and union perception (*β* = 0.269, *p* < 0.001), which captures 14.7% of the observed variance (*r* ^2^ = 0.147). The predictive strength shifts notably in the second model; here, the inclusion of union expectation drives the association higher (*β* = 0.396, *p* < 0.001) and pushes the explained variance to 26.3% (*r* ^2^ = 0.263). A final combined analysis demonstrates that while both wage satisfaction (*β* = 0.192, *p* < 0.001) and union expectation (*β* = 0.344, *p* < 0.001) remain significant, they collectively account for 33.3% of the variance in union perception (*r* ^2^ = 0.333). While both variables clearly matter, union expectation emerges as the more potent driver in this relationship.

**TABLE 5:** Associations of wage satisfaction with union perception and union expectation: Regression and mediation analysis.

Dependent variable	Model	Independent variable (Path)	*β*	SE	*t*	*r*	*r* ^2^	*F*	*p*	DW (1.5‐2.5)	Effect type	95% CI (LL–UL)
Union Perception (Mediator)	1	Wage Satisfaction (a)	0.269	0.045	5.979	0.383	0.147	35.754	< 0.001	1.242	Direct (X → M)	0.180–0.357
Union Expectation	1	Wage Satisfaction (c: total effect)	0.224	0.061	3.656	0.246	0.060	13.369	< 0.001	2.014	Total (X → Y)	0.103–0.344
	2	Union Perception (b)	0.637	0.084	7.614	0.513	0.266	37.505	< 0.001	2.187	Direct (M → Y)	0.472–0.801
	3	Wage Satisfaction (c′)	0.053	0.059	0.896	—	0.266	37.505	0.371	2.197	Direct (X → Y)	−0.063 – 0.168
		Union Perception	0.637	0.084	7.614	—	—	—	< 0.001	—	Direct (M → Y)	0.472–0.801
Mediation (PROCESS Bootstrap, 5000 resamples)	—	Indirect Effect (a × b)	0.171	0.044	—	—	—	—	—	—	Indirect (X → M → Y)	0.095–0.263

*Note:* *N* = 210. Mediation analysis was conducted using Hayes' PROCESS macro (Model 4) with 5000 bootstrap samples. Statistical significance was evaluated at *p* < 0.05. The indirect effect is considered significant when the bootstrap confidence interval does not include zero.

Abbreviations: CI, confidence interval; DW, Durbin–Watson statistic; LL, lower limit; UL, upper limit.

Three regression stages evaluated how various factors drive union expectations. Initial results show that wage satisfaction maintains a significant link to expectations (*β* = 0.224, *p* < 0.001), even though it accounts for a modest 6% of the total variance (*r* ^2^ = 0.060). However, this dynamic shifts entirely with the introduction of union perception. This second model reveals a much more robust relationship (*β* = 0.637, *p* < 0.001), pushing the explained variance up to 26.6% (*r* ^2^ = 0.266). In the final integrated model, union perception holds its strong effect (*β* = 0.637, *p* < 0.001), yet wage satisfaction effectively collapses as a predictor (*β* = 0.053, *p* = 0.371). Because the explanatory power stays flat at 26.6% (*r* ^2^ = 0.266), the data suggests that union perception absorbs the initial impact of wages, rendering wage satisfaction a redundant factor in predicting expectations. Collectively, these results demonstrate that union perception plays a decisive role in shaping nurses' expectations from unions, whereas wage satisfaction does not exert a significant direct effect once union perception is accounted for (Table 5).

**FIGURE 2:**
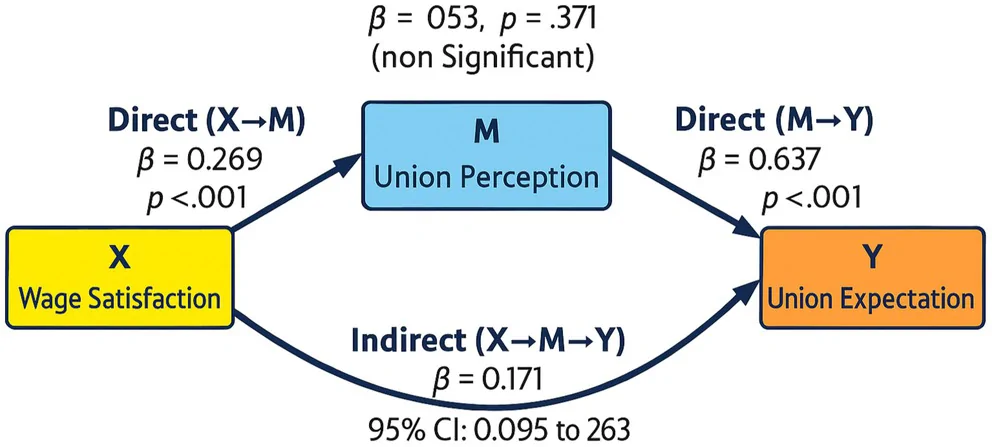
Mediation model results between wage satisfaction, union perception, and union expectation.

Figure 2 illustrates the mediation model tested with Hayes' PROCESS macro (Model 4). The analysis revealed that wage satisfaction significantly predicted union perception (*β* = 0.269, *p* < 0.001), which in turn exerted a strong and significant effect on union expectation (*β* = 0.637, *p* < 0.001). The direct path from wage satisfaction to union expectation was not statistically significant (*β* = 0.053, *p* = 0.371), suggesting the absence of a direct effect. However, the indirect effect of wage satisfaction on union expectation through union perception was statistically significant (*β* = 0.171), with a 95% bootstrap confidence interval excluding zero (0.094–0.263). This provides robust evidence of full mediation, whereby wage satisfaction affects union expectation exclusively through its effect on union perception.

Overall, the mediation analysis highlights the central role of union perception as a psychological mechanism linking wage satisfaction to union‐related expectations. These results imply that even though wage dissatisfaction is a salient issue for nurses, their expectations from unions are not directly shaped by wages themselves but rather by how wages affect their broader perception of unions as representative organisations (Figure 2).

### 5.3 Qualitative Results

Four explanatory themes emerged from the qualitative data to better understand the relationship between nurses' wage satisfaction and union membership. These themes are: wages and working conditions, the role and impact of unions, perceptions of unions, and expectations from unions.

#### 5.3.1 Theme 1. Wages and Working Conditions

The majority of participants expressed that nurses' wages do not reflect the quality of their work and that they struggle to make ends meet. One participant summarised this by saying, ‘The wage nurses receive is far below the labour they put in. We do a heavy job, but it's not worth it’. Additionally, issues such as heavy workloads and burnout were identified as major concerns. Participants reported feeling physically and psychologically strained due to overtime, shift irregularities, and staffing shortages. One participant noted, ‘Our shifts are very busy, sometimes we can't even spend enough time with a patient. We are constantly running, but we're not paid for our labour’. Furthermore, the lack of personal rights for nurses was highlighted as a factor negatively impacting their professional satisfaction. One participant explained, ‘Our leave periods are very limited, additional payments are irregular, and opportunities for professional advancement are almost non‐existent. If these issues aren't addressed, it will be hard to stay in this profession’.

#### 5.3.2 Theme 2. The Role and Impact of Trade Unions

The role of trade unions in defending the rights of healthcare workers was critically assessed by the participants. Those who felt unions were not effective enough in the fight for better wages noted that union activities failed to produce tangible changes on the ground. One participant remarked, ‘Unions only make statements, but nothing changes in reality. Our wages are still low, and conditions are harsh. We don't see any real effort’. Another frequent criticism was the lack of independence and the political influence over unions. One participant emphasised the need for unions to be independent from government control, stating, ‘Unions should be independent, but in our country, unions operate under political pressure. Unions that derive their power from politics, not from workers, cannot protect us’. However, some participants acknowledged that unions do advocate for workers' rights through demonstrations, press releases, and legal actions, although they emphasised that these efforts should be more systematic and results‐driven. As one participant explained, ‘Yes, unions are fighting, but our hands are tied. We can't get stronger without support from employees’.

#### 5.3.3 Theme 3. Perception of Trade Unions

Nurses' perceptions of unions are largely characterised by distrust. The majority of participants expressed that unions fail to adequately protect the rights of their members, leading to a decline in employees' faith in unions. One participant commented, ‘They don't even know what unionism means. Membership is just on paper’. Another significant issue raised was the lack of communication between unions and their members. One participant explained, ‘I don't know what I will gain by becoming a member of the union. There's no information, no communication. Being a member doesn't change anything’. The management style of unions also drew criticism. One participant suggested that merit should be the primary focus in union leadership, stating, ‘There should be no favouritism in union management. Leaders should genuinely listen to the demands of employees’. Another participant further emphasised the problems in leadership and democratic decision‐making, stating, ‘In our union, the same individuals have held leadership positions for years, prioritising their own interests over those of the members, and it is almost impossible to be informed about internal processes or to know how membership fees are used’.

#### 5.3.4 Theme 4. Expectations From Trade Unions

The primary expectation from trade unions is a wage increase and improvements in personal rights. The majority of participants expressed that unions should intensify their efforts in advocating for better wages: ‘Our expectation is simple: Better wages, better working conditions’. However, union expectations go beyond material gains. Workers also stressed the importance of unions being more independent and transparent: ‘Unions should stay out of politics. They should defend the rights of their members, not special interest groups’. A recurring theme was the need for stronger communication between unions and employees. One participant noted, ‘Unions should engage with us more; they should be here to truly understand our problems, not just to collect dues’.

### 5.4 Integration of Quantitative and Qualitative Findings

The integration of quantitative and qualitative findings clarifies how wage satisfaction, union perception, and union expectations interact within nurses' professional experiences. Regression and mediation analyses demonstrated that wage satisfaction significantly predicted union perception (*β* = 0.269, *p* < 0.001) but did not exert a significant direct effect on union expectation (*β* = 0.053, *p* = 0.371). Instead, union perception emerged as the central explanatory mechanism, exerting a strong effect on union expectations (*β* = 0.637, *p* < 0.001) and fully mediating the relationship between wage satisfaction and expectation (indirect effect *β* = 0.171, 95% CI [0.095, 0.263]).

Qualitative findings provide substantive explanations for these statistical relationships. Under Theme 1 (Wages and Working Conditions), participants consistently emphasised that their wages do not reflect the intensity and burden of their work, a perception that aligns with the low mean wage satisfaction reported in the descriptive analysis (M = 1.94; Table 4). However, these narratives also reveal that dissatisfaction with wages alone does not automatically generate expectations from unions, directly supporting the nonsignificant wage satisfaction → union expectation path observed in the quantitative analysis.

Themes.

2 (Role and Impact of Trade Unions) and 3 (Perception of Trade Unions) illuminate why union perception functions as a mediating mechanism. Participants frequently criticised unions for political entanglements, weak communication, and lack of transparency, all of which directly correspond to the strong predictive effect of union perception on union expectations (*β* = 0.637). These accounts demonstrate that nurses' expectations are shaped not simply by economic dissatisfaction, but by how unions are evaluated as credible, independent, and effective representatives.

Finally, Theme 4 (Expectations from Trade Unions) clarifies the content of these expectations. While demands for better wages and working conditions remain salient, participants emphasised transparency, merit‐based leadership, and stronger communication as equally important. This pattern is consistent with the increase in explained variance from *R* ^2^ = 0.060 to *R* ^2^ = 0.266 when union perception is included in the model, indicating that expectations are driven primarily by perceptions of union governance rather than wages alone.

Overall, the integration reveals a convergent mixed‐methods pattern: wage dissatisfaction is widespread but insufficient to shape union expectations unless filtered through nurses' perceptions of unions. Nurses' narratives demonstrate that the credibility, independence, and communication practices of unions determine whether dissatisfaction transforms into concrete expectations for change. Thus, the mixed‐methods evidence highlights that union perception operates not only as a mediating statistical construct, but also as a lived evaluative experience bridging nurses' frustrations with their aspirations for professional representation (Table 6).

**TABLE 6:** Joint display of quantitative and qualitative findings on wage satisfaction, union perception, and union expectations.

Quantitative finding	Qualitative theme	Representative quote	Integration/Interpretation
Wage satisfaction significantly predicts union perception (*β* = 0.269, *p* < 0.001)	Theme 1. Wages and Working Conditions	‘The wage nurses receive is far below the labour they put in. We do a heavy job, but it's not worth it.’	Wage dissatisfaction shapes how nurses evaluate unions, supporting the significant path from wage satisfaction to union perception. Dissatisfaction with pay does not directly translate into expectations but influences perceptions of unions as potential representatives.
Wage satisfaction does not directly predict union expectation (*β* = 0.053, *p* = 0.371)	Theme 1. Wages and Working Conditions	‘We're constantly running, but we're not paid for our labour’.	Although wage dissatisfaction is intense, these narratives show that dissatisfaction alone is insufficient to generate union expectations, consistent with the nonsignificant direct effect.
Union perception strongly predicts union expectation (*β* = 0.637, *p* < 0.001)	Theme 3. Perception of Trade Unions	‘They don't even know what unionism means. Membership is just on paper’.	Negative perceptions of unions directly reduce expectations, whereas stronger and more positive perceptions increase expectations, mirroring the strong and significant predictive effect of perception on expectation.
Indirect effect of wage satisfaction on union expectation through union perception is significant (*β* = 0.171, 95% CI [0.095, 0.263])	Theme 2. Role and Impact of Trade Unions Theme 4. Expectations from Trade Unions	‘Unions should stay out of politics. They should defend the rights of their members, not special interest groups’.	This quotation directly reflects the mediating mechanism: wage dissatisfaction influences expectations only when filtered through perceptions of union independence, transparency, and effectiveness.
Explained variance in union expectation increases from *R* ^2^ = 0.060 (wage satisfaction only) to *R* ^2^ = 0.266 (with union perception included)	Theme 4. Expectations from Trade Unions	‘Our expectations are simple: better wages, better working conditions… but also transparency and communication’.	Expectations are shaped less by wages alone and more by how nurses perceive unions' responsiveness and governance, consistent with the substantial increase in explained variance after adding perception.

## 6 Discussion and Theoretical Implications

This study, which explored the relationship between nurses' wage satisfaction, union perceptions, and union expectations, revealed that union participation is a multifaceted and complex phenomenon that cannot be understood solely in economic terms. The findings show that while wage satisfaction was positively associated with union perception, it is the perception of the union that demonstrated a stronger association with union expectations. This suggests that nurses' expectations of unions extend beyond material demands, such as wage increases, to include structural concerns, such as organisational transparency, political independence, and greater participation in decision‐making processes.

This shift in union dynamics is also reflected in international literature. Historically, unionism focused on wage negotiations and improving working conditions (Freeman and Medoff 1984). However, today, union commitment is increasingly related to factors such as organisational representativeness, governance transparency, and active member involvement in decision‐making (Blanchflower et al. 2022). This transformation can be attributed to the evolving role of unions, which are no longer seen merely as instruments of self‐interest but also as embodiments of collective identity and moral representation. Wilkinson et al. (2020) argue that contemporary union legitimacy is grounded in normative values such as ethical representation, justice, and democratic participation. Rather than reiterating empirical findings, the present study contributes to this literature by situating nurses' union‐related attitudes within this broader normative turn in contemporary union theory.

Our study's finding that union perception was strongly associated with union expectations indicates that nurses' attitudes toward unions are largely shaped by their subjective experiences and evaluations of union functioning. This aligns with Freeman and Medoff's (1984) ‘voice’ theory, which posits that unions serve as a platform for workers to express their concerns. However, the findings extend this framework by demonstrating that ‘voice’ is not experienced merely as the opportunity to express dissatisfaction, but as a normative evaluation of whether unions are perceived as transparent, representative, and independent. In the Turkish context, nurses appear to view unions not only as defenders of economic interests but also as institutions expected to represent professional identity and autonomy (Dierkes et al. 2024; Cake 2024).

Qualitative findings further clarify why this evaluative process is decisive. Participants frequently expressed frustration with exclusion from decision‐making processes and mistrust stemming from limited transparency, suggesting that unions are perceived as falling short of fulfilling their representative role. This resonates with the broader ‘crisis of union representation’ discussed in the literature (Gumbrell‐McCormick and Hyman 2013). A critical distinction exists between the statistical links identified and the logic behind them. Although regression analysis confirms a correlation between wage satisfaction and union perception, the specific drivers of this relationship remain outside the scope of raw numbers. Rather, the study identifies core mechanisms—specifically transparency, political independence, and representational strength—through an inductive analysis of the qualitative data. This dual‐method approach allows the nuanced themes extracted from interviews to ground the broader patterns observed in the quantitative models. This distinction clarifies how different components of the findings contribute to theory building.

In Türkiye, this representational crisis appears particularly pronounced in the health sector, where unions are often perceived as politically aligned rather than member‐oriented (Korkmazer 2021; Kuru 2022). The qualitative data strongly support this interpretation, with participants criticising the ineffectiveness and low visibility of union activities. These criticisms clarify why wage dissatisfaction, on its own, failed to translate directly into union expectations in the quantitative analysis; expectations took shape only when dissatisfaction passed through nurses' positive or negative judgements about union credibility. Additionally, disengagement among more experienced nurses was frequently linked to professional burnout and labour fatigue (Tosun 2023), suggesting that emotional and occupational strain further conditions how unions are evaluated.

The findings also challenge the assumptions of rational choice approaches to union participation (Freeman and Medoff 1984). Despite low wage satisfaction, union expectations remained relatively high, indicating that union engagement cannot be explained solely by cost–benefit calculations. Instead, normative considerations—such as institutional trust, fairness, and democratic governance—emerged as central evaluative criteria. This supports prior research highlighting the importance of organisational commitment and institutional trust in shaping union‐related attitudes (Fiorito et al. 2010).

Kelly's (2012) mobilisation theory offers a complementary perspective by emphasising the role of collective identity, justice, and moral legitimacy in union consciousness. The present findings suggest that mobilisation potential depends less on the intensity of material grievances and more on whether unions are perceived as legitimate vehicles for collective representation. This helps explain why participants simultaneously expressed strong expectations from unions and deep dissatisfaction with their current performance. Strengthening union effectiveness, therefore, requires not only addressing economic demands but also rebuilding social capital through transparency, inclusiveness, and credible representation (Heery 2010; Hodder and Edwards 2015).

Gender did not serve as a focal variable in the regression or mediation analyses; however, descriptive patterns pointed to slightly higher levels of union perception and economic expectations among male nurses. Qualitative evidence helped to situate this difference. Female nurses referred to heavy workloads, unequal caregiving responsibilities, and persistent gender norms that associate leadership with men, all of which foster feelings of distance and misrepresentation within unions. Previous studies likewise document how male‐dominated union structures and organisational cultures limit women's participation and access to leadership roles, thereby reproducing gendered inequalities in union democracy (Urhan 2017; Keleş 2018). Taken together, these dynamics indicate that gender affects union perceptions indirectly through structural and normative constraints.

Taken together, the study's theoretical contribution does not rest on introducing a novel analytical model, but on advancing a conceptual insight grounded in both empirical evidence from regression and mediation analyses and inductively generated qualitative findings. The results position wage dissatisfaction as a relevant background condition, while showing that nurses form union expectations mainly through normative judgements about unions' credibility, independence, transparency, and capacity for representation. In this respect, union perception functions not simply as a statistical mediator but as a socially situated evaluative process that channels workplace grievances into expectations for collective voice and representation. Broader research further supports this interpretation by linking union engagement not only to instrumental gains but also to well‐being, trust, and democratic orientations, highlighting the broader importance of perception‐based union legitimacy (Blanchflower et al. 2022).

## 7 Limitations

The study is subject to several methodological limitations. A primary challenge involves the lack of current national reports on the nursing workforce and remuneration in Türkiye, a gap that complicates the effort to contextualise the structural forces driving wage satisfaction and union attitudes. Without this broader framework, the study's focus on a single institutional network becomes even more significant. While the participants represent various clinical units, they operate under a shared administrative culture and a specific socio‐economic backdrop. This organisational uniformity restricts the findings' external validity, suggesting that these conclusions may not apply to healthcare settings with different labour relations or regional governance structures.

Participant representation also introduces specific limitations. The qualitative phase relied heavily on union leaders and active members, while non‐unionised nurses—especially those sceptical of or disengaged from organised labour—remained underrepresented. Consequently, the analysis may miss the more critical or marginalised perspectives within the profession.

Finally, although gender‐related patterns emerged during qualitative and descriptive stages, the regression and mediation models did not include gender as a core focal variable. This absence prevents a rigorous causal interpretation of how gender differences affect union expectations. Future research should more explicitly integrate gender to examine how organisational structures and professional roles shape collective bargaining attitudes.

To advance this line of inquiry, future research should consider larger, multi‐site samples across different cities with varying living standards and working conditions, as well as cross‐national replications. Such designs would allow for the exploration of how wage satisfaction, union perceptions, and expectations evolve across diverse organisational and socio‐cultural contexts. In addition, this study did not provide an in‐depth examination of participants' individual union experiences, reasons for leaving unions, or sector‐specific dynamics of union organising, all of which warrant further investigation. Because the study was confined to the Turkish context, cross‐national comparisons should be made with caution due to cultural and structural differences.

## 8 Conclusion and Policy Recommendations

This study demonstrates that although nurses' wage satisfaction is generally low, higher wage satisfaction is nonetheless associated with more favourable perceptions of unions. More importantly, union perception emerged as a strong predictor of union expectations, underscoring its mediating role in shaping nurses' engagement with unions. These findings indicate that union participation cannot be explained solely by economic considerations, but is also driven by normative factors such as representativeness, transparency, political independence, and institutional trust. Nurses thus evaluate unions not merely as transactional bargaining agents, but as organisations expected to embody professional identity, fairness, and democratic accountability.

From a policy perspective, strengthening nurses' engagement with unions requires organisational reforms that go beyond wage‐related advocacy. Unions should adopt more transparent, participatory, and merit‐based governance structures, reduce perceived political entanglements, and enhance direct communication with members at the grassroots level. Such measures are likely to reinforce perceptions of legitimacy and credibility, thereby increasing nurses' expectations and willingness to engage. At the same time, wage policies that ensure fairness and sustainability remain essential for improving baseline satisfaction and addressing the material conditions underlying discontent.

In conclusion, union participation among nurses should be understood as a multifaceted phenomenon shaped by the interaction of economic conditions and normative evaluations. Sustainable union engagement depends on balancing material advocacy with ethical governance and democratic representation, enabling unions to function as credible platforms for professional voice, collective trust, and long‐term workforce sustainability.

## Author Contributions

S.T., G.C., and D.B.S.: Made substantial contributions to conception and design, or acquisition of data, or analysis and interpretation of data; Involved in drafting the manuscript or revising it critically for important intellectual content; Given final approval of the version to be published. Each author should have participated sufficiently in the work to take public responsibility for appropriate portions of the content; Agreed to be accountable for all aspects of the work in ensuring that questions related to the accuracy or integrity of any part of the work are appropriately investigated and resolved.

## Funding

The authors have nothing to report.

## Conflicts of Interest

The authors declare no conflicts of interest.

## Supporting information


**Data S1:** jan70580‐sup‐0001‐Supinfo1.docx.

## Data Availability

Research data are not shared.
